# Development of orthogonal aminoacyl-tRNA synthetase mutant for incorporating a non-canonical amino acid

**DOI:** 10.1186/s13568-024-01706-3

**Published:** 2024-05-24

**Authors:** Dongheon Lee, Ja Gyung Kim, Tae Wan Kim, Jong-il Choi

**Affiliations:** Department of Biotechnology and Bioengineering, Chonnam National University, Gwangju, 61186 Republic of Korea

**Keywords:** Non-canonical amino acid, *Methanosaeta concilii*, Aminoacyl-tRNA synthetase, Para-azido-L-phenylalanine, Amber suppression

## Abstract

Genetic code expansion involves introducing non-canonical amino acids (ncAAs) with unique functional groups into proteins to broaden their applications. Orthogonal aminoacyl tRNA synthetase (aaRS), essential for genetic code expansion, facilitates the charging of ncAAs to tRNA. In this study, we developed a new aaRS mutant from *Methanosaeta concilii* tyrosyl-tRNA synthetase (Mc TyrRS) to incorporate para-azido-l-phenylalanine (AzF). The development involved initial site-specific mutations in Mc TyrRS, followed by random mutagenesis. The new aaRS mutant with amber suppression was isolated through fluorescence-activated cell sorting. The *M. concilii* aaRS mutant structure was further analyzed to interpret the effect of mutations. This research provides a novel orthogonal aaRS evolution pipeline for highly efficient ncAA incorporation that will contribute to developing novel aaRS from various organisms.

## Introduction

Non-canonical amino acids (ncAAs) with unique functional groups can diversify protein structure and activity, overcoming the structural or functional limitations of canonical amino acids. In particular, the site-specific binding of ncAA is currently researched in synthetic biology, proteomics, and medicine (Bain et al. 1989). As an example, introducing ncAAs is garnering attention as an innovative method for increasing the half-life of therapeutic proteins. Contrasting PEGylation via cysteine or lysine, incorporating site-specific ncAAs with certain residues allows site-specific PEGylation and large-scale synthesis of homogeneous proteins (Nguyen et al. 2009; Yang et al. 2016).

Genetic code expansion is a representative method to incorporate ncAAs by constructing novel translation systems that do not cross-act with the host cell. The vital elements of this technology are (1) a codon capable of encoding ncAA (a stop codon or a quadruplet codon), (2) a tRNA that recognizes the codon (suppressor tRNA), and (3) an aminoacyl-tRNA synthetase (aaRS) that aminoacylates the ncAA to the tRNA (Wang et al. 2001). An essential factor is that the tRNA, aaRS, and ncAA must work orthogonally to avoid interfering with the host cell's translation system.

Therefore, aaRS/tRNA pairs were imported from several organisms into *Escherichia coli* to fulfill these requirements. For example, the tyrosyl-tRNA synthetase/tRNA^Tyr^ pair from *Methanococcus jannaschii* (Wang et al. 2001) or *Archaeoglobus fulgidus* (Cervettini et al. 2020) and the pyrrolysyl-tRNA synthetase/tRNA^Pyl^ pair from *Methanosarcina barkeri* (Srinivasan et al. 2002) or *Methanosarcina mazei* (Odoi et al. 2013) were developed to incorporate ncAA into proteins in *E. coli*. Although several mutant aaRS/tRNA pairs were developed, additional aaRS/tRNA pairs are still being required to broaden the usage of ncAAs. However, most aaRSs used to incorporate ncAAs are derived from *M. jannaschii* (Chin et al. 2002a, 2002b; Deiters and Schultz 2005; Guo et al. 2008; Schultz et al. 2006; Xie et al. 2004; Young et al. 2010), and new aaRS from unprecedented sources are needed for the universalization of genetic code expansion.

Herein, we introduce a new aminoacyl-tRNA synthetase mutant from *Methanosaeta concilii* for para-Azido-L-phenylalanine (AzF) incorporation. *M. concilii* aaRS was selected after searching homology with the Basic Local Alignment Search Tool (BLAST). From the previous report on the orthogonal *M. jannaschii* tyrosyl-tRNA synthetase mutant, *M. concilii* aaRS was mutated at specific sites to disfavor natural substrates. An additional random mutation was conducted to favor ncAA, and a new *M. concilii* aaRS with amber suppression was isolated through fluorescence-activated cell sorting (FACS). Also, structural model was predicted to elucidate the effect of mutations. We expect this study could broaden the pool of orthogonal aminoacyl-tRNA synthetase for genetic code expansion.

## Materials and methods

### Strains and media

*E. coli* DH10B (Invitrogen, Waltham, MA) was used for plasmid cloning and recombinant protein expression. LB media (BD-Difco, Franklin Lakes, NJ) was utilized as the culture media. The working antibiotic concentrations were as follows: 50 μg/mL chloramphenicol (Daejung Chemicals, Siheung, Republic of Korea) and 50 μg/mL gentamicin (KisanBio, Seoul, Republic of Korea). Primers used in this study were synthesized and purchased from Macrogen (Seoul, Republic of Korea). AzF (Chem-Impex, Wood Dale, IL), a non-canonical amino acid, was included as a substrate for aaRS. Arabinose (Alfa Aesar, Haverhill, MA) and isopropyl β-d-1-thiogalactopyranoside (IPTG, Bioneer, Daejeon, Republic of Korea) were applied as inducers.

### Selection of aaRS from new resource

BLAST was employed on the NCBI database to align mutant tyrosyl-tRNA synthetase genes of *M. jannaschii* (Mj TyrRS), which was previously developed to introduce non-canonical amino acids (Chin et al. 2002b).

### Site-directed mutagenesis of Mc TyRS

From previous reports on the Mj TyrRS mutant with amber suppression, specific sites in proteins are known to be essential for substrate recognition. Mj TyrRS and Mc TyrRS protein sequences were aligned with the pairwise sequence alignment of EMBL-EBI (Madeira et al. 2022) to identify the significant sites in Mc TyrRS, and visualization was performed with ESPript 3 (Robert and Gouet 2014). Tyrosine 33, aspartic acid 162, and lysine 166 in Mc TyrRS were selected for site-directed mutation. Rationally designed genes were synthesized by Genophile (Gwangju, Republic of Korea).

### Random mutagenesis for library construction

Random mutagenesis was conducted using the Genemorph II random mutagenesis kit (Agilent, Santa Clara, CA) according to the manufacturer's instructions. DNA fragments for library creation were cloned into *Bgl*II/*Pst*I sites of the plasmid backbone. The plasmid backbone was generated via PCR on the pEVOL-pAzF plasmid (Addgene: 31186), including suppressor tRNA under the proK promoter, the chloramphenicol resistance gene under the CAT and aaRS gene under ara promoter (Lee et al. 2023). As a result, the pEVOL-McTyrRSmut plasmid was constructed for mutant McTyrRS expression under the ara promoter. The amplified plasmid backbone was confirmed using Sanger sequencing.

### sfGFP expression with the amber codon

Superfolder Green Fluorescence Proteins (sfGFPs) bearing the TAG codon at position 204 were expressed with the aaRS mutant library to quantify aaRS activity (Kim and Choi 2022). In previous study, several positions in sfGFPs were tested to bear TAG codon. Among them, sfGFPs mutated at position 204 showed the intended fluorescence. In this system, the presence of the amber codon (TAG) at the midpoint of the sfGFP sequence plays a critical role. If an amino acid is not incorporated at the amber codon site due to the lack of a compatible aminoacyl-tRNA synthetase, translation is terminated early, resulting in a truncated sfGFP that does not exhibit fluorescence. Conversely, successful incorporation of the AzF allows for full-length sfGFP synthesis, rendering the protein fluorescent. *E. coli* DH10B was co-transformed with the pEVOL-McTyrRSmut plasmid and pSEVA631pt-sfGFP204amb, including the sfGFP gene with the TAG codon under the Tac promoter and a gentamicin resistance gene under the Pc promoter.

As a control, pEVOL-pAzF or pEVOL-McTyrRSwt were co-transformed with pSEVA631pt-sfGFP204amb and cultured under the same conditions. A single colony was pre-cultured in 5 mL LB media supplemented with antibiotics at 37 °C overnight. Pre-cultured cells were inoculated into 50 mL LB media supplemented with antibiotics, 0.5 mM AzF, 0.2% (w/v) arabinose, and 0.5 mM IPTG at an initial OD_600_ nm = 0.1, and cultured at 37 °C for 9 h. Experiments were performed three times, and standard deviations were presented.

### Microscope

sfGFP expressed culture was centrifuged to prepare the sample. Collected cell was deposited on a glass slide and covered by cover glass. Each specimen was observed at 1000× magnification by ZEISS Axio Scope.A1 Microscope (ZEISS, Oberkochen, Baden-Württemberg, Germany) and Microscope illuminating system-HBO 100 (ZEISS).

### Microplate reader

Optical density (600 nm) and fluorescence intensity (excitation 485 nm, emission 528 nm) were measured in 96-well black clear-bottom plates (SPL Life Science Co., Gyeonggi-do, South Korea) with a Synergy H1 Hybrid Multi-Mode Reader (BioTek, Winooski, VT). Fluorescence intensity was normalized by dividing the fluorescence value by the optical density value.

### Fluorescence-activated cell sorting (FACS)

The sfGFP-expressed cell culture with the random mutagenesis library was diluted to OD_600_ nm = 1, centrifuged, and washed twice with 1 mL of 1 × phosphate-buffered saline (PBS). MoFlo XDP Flow Cytometer & Sorter (Beckman Coulter, Brea, CA) sorted cells with the top 1 ~ 3% fluorescence for three rounds.

### Prediction of the mutant Mc TyrRS structure

Mc TyrRS wt, Mc TyrRS mutant 6, and mutant Mc TyrRS protein sequences were submitted to the SWISS-MODEL server for homology modeling (Waterhouse et al. 2018). Tertiary structures were predicted based on the crystal structure of Mj TyrRS (PDB: 1J1U) as a template. Docking for AzF was conducted with AutoDock Vina (v.1.1.2) (Eberhardt et al. 2021). Side chains near the substrate around 5 Å were specified as flexible for recognizing substrate. The figure’s tertiary structure was prepared using PyMOL (v2.5.2) (Schrodinger 2021).

## Results

### Rational evolution of Mc TyrRS

For the development of new aminoacyl-tRNA synthetase for AzF incorporation, BLAST was utilized on the NCBI database to identify new resources. This method aligned the previously developed tyrosyl-tRNA synthetase mutant of *M. jannaschii* (Mj TyrRS), which was developed for introducing AzF. From this alignment, tyrosyl-tRNA synthetase of *Methanosaeta concilii* GP6 (Mc TyrRS) (Accession number: WP_013718185.1) was selected for further development as it presented the highest similarity of 50% identities and 73% positives compared to the Mj TyrRS mutant.

**Fig. 1:**
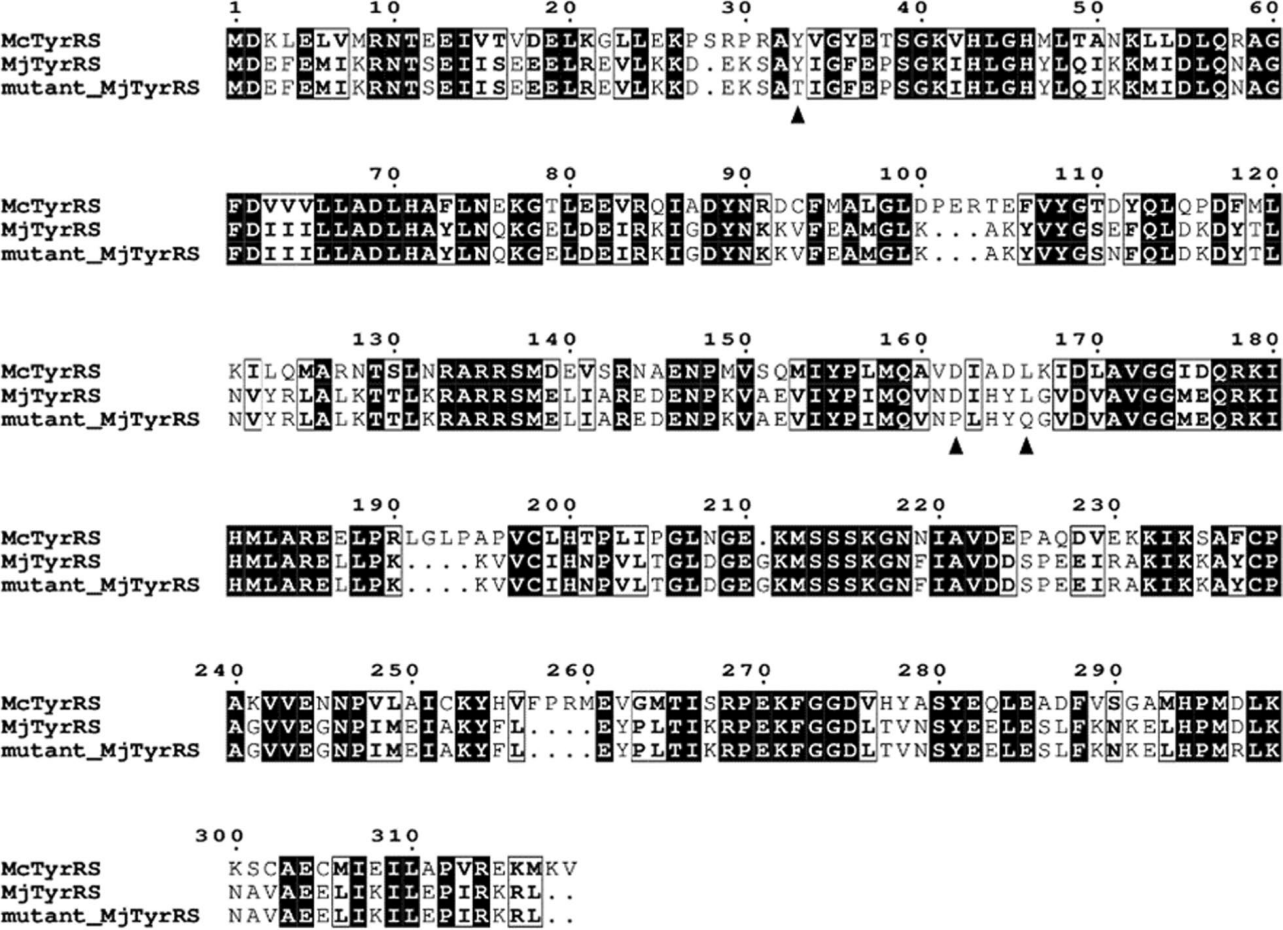
Alignment for selecting target residues. Mj TyrRS and Mc TyrRS protein sequences were aligned with local alignment, preserving conserved sequences. Target residues for rational design (Y33, D162, and L166 of Mc TyrRS corresponding to Y32, D158, and L162 of Mj TyrRS) were presented as a triangle

Based on previous research about the development of Mj TyrRS mutants with amber suppression and orthogonality, five mutated Mj TyrRS residues, Y32, E107, D158, I159 and L162, were identified (Chin et al. 2002b). Protein sequence of Mc TyrRS was aligned to Mj TyrRS and conformed that each residue was corresponded to Y33, D111, D162, I163, and L166, respectively. From the Mj TyrRS mutants and Mc TyrRS alignment, three sites (Y33, D162, and L166) in Mc TyrRS were selected for target of mutation as they were crucial for synthesizing charged tRNA with AzF (Fig. 1).

Several Mc TyrRS mutants were constructed through site-directed mutagenesis. The Mc TyrRS mutant 4 was mutated with Y33L, D162L, and L166Q; Mc TyrRS mutant 5 with Y33A, D162V, and L166D; Mc TyrRS mutant 6 with Y33G and D162T; and Mc TyrRS mutant 7 with Y33L, D162Q, and L166S. sfGFP with an amber codon at position 204 was co-expressed and observed through fluorescence microscope to verify mutant activity (data not shown). Recombinant *E. coli* harboring Mc TyrRS wt expressed sfGFP in the absence or presence of AzF. On the other hand, recombinants harboring Mc TyrRS mutants 4 ~ 6 exhibited no or little fluorescence with AzF. Among them, Mc TyrRS mutant 6 showed higher fluorescence with AzF compared to no AzF. Therefore, it was selected for further development to charge AzF.

### Random mutagenesis and sfGFP expression

**Fig. 2:**
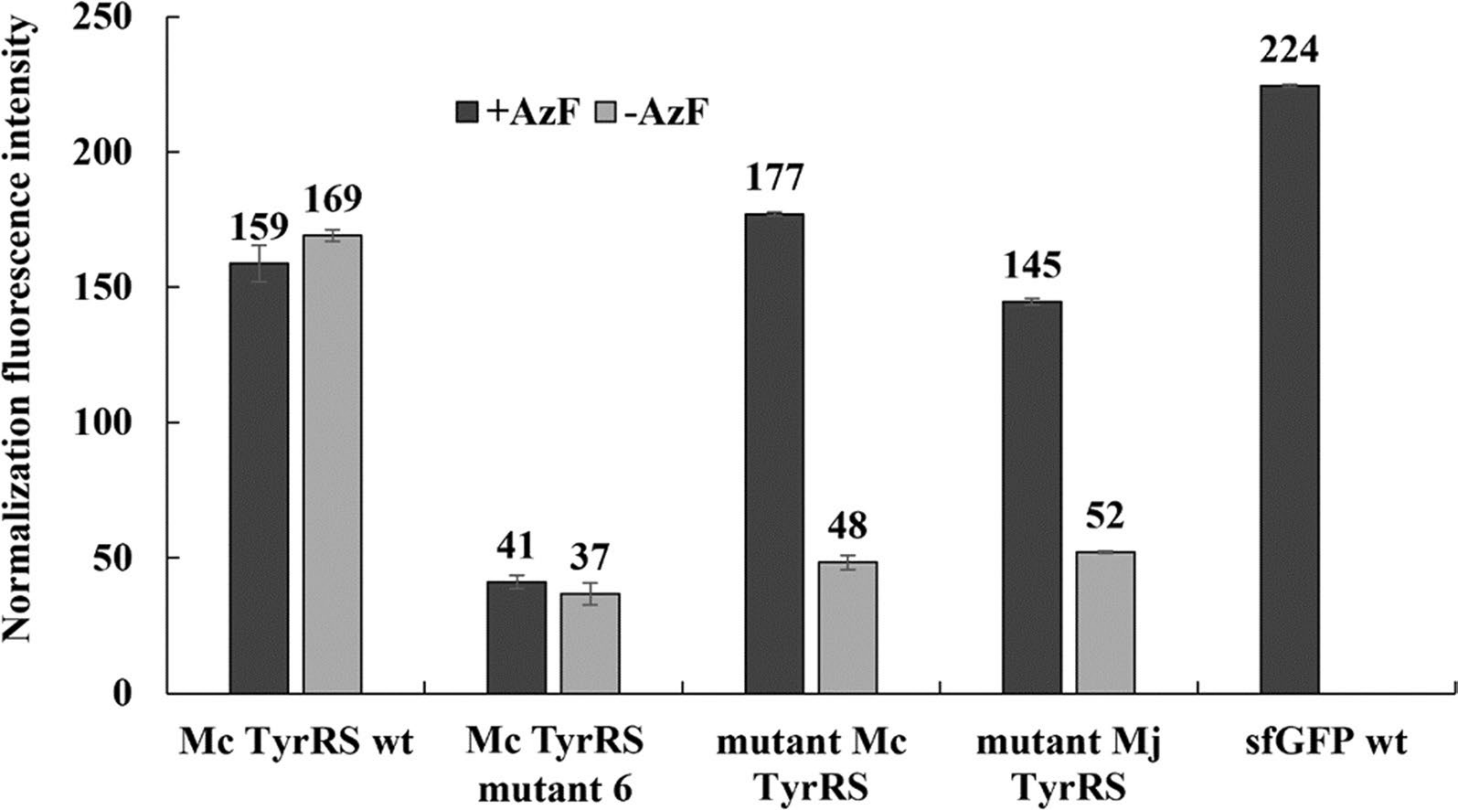
Comparison of amber suppression efficiency. Normalized fluorescence intensity was calculated by dividing fluorescence by OD 600 nm to determine the suppression efficiency of each aaRS. Mutant Mc TyrRS screened from a random mutagenesis library presented selectivity in AzF recognition, while Mc TyrRS wt and Mc TyrRS mutant 6 did not. n = 3; error bars, mean ± s.d

For further development, random mutagenesis was performed on the Mc TyrRS 6 mutant to establish variants capable of amber suppression with AzF. Cells harboring mutant libraries from random mutagenesis and the sfGFP gene with an amber codon were screened using FACS over three rounds. Sorted factions were spread on LB agar plates with antibiotics, and single colony selection using sfGFP expression was performed successively. As a result, mutant Mc TyrRS was isolated and sfGFP was expressed to confirm the activity (Fig. 2). The activity of aaRS was confirmed with normalized fluorescence intensity. Normalized fluorescence intensity of recombinant with mutant Mc TyrRS was 177 and 48 in the presence and absence of AzF, respectively, compared to Mc TyrRS wt was 159 and 169 or Mc TyrRS mutant 6 was 41 and 37.

### 3D structure analysis of mutant Mc TyrRS

**Fig. 3:**
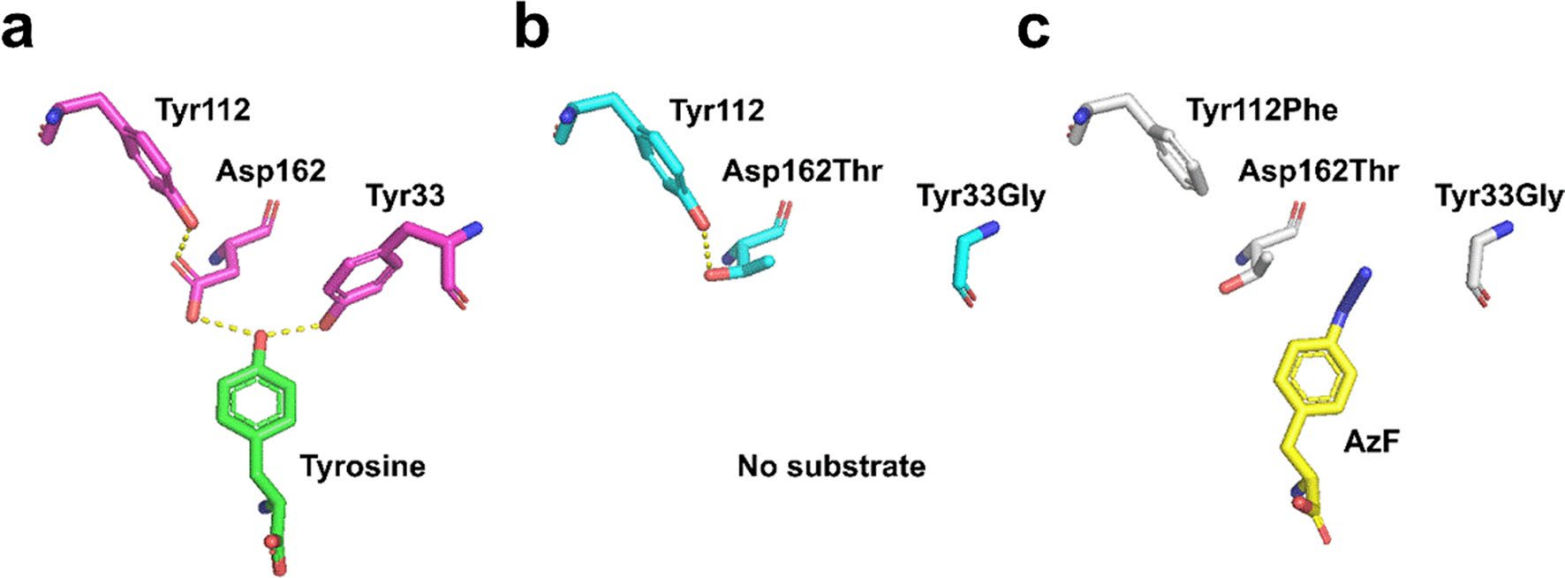
Predicted structures of (**a**) Mc TyrRS wt, (**b**) Mc TyrRS mutant 6, and (**c**) mutant Mc TyrRS. Three residues in the active sites (Y33G, Y112F, and D162T) and substrate (tyrosine or AzF) were represented with modeling. Compared to Mc TyrRS wt charged the tyrosine as substrate, Mc TyrRS mutant 6 (Y33G, D162T) disfavored not only tyrosine, but also AzF. With the additional mutation in mutant Mc TyrRS (Y33G, Y112F, and D162T), it could recognize the AzF. The yellow dotted line represents a hydrogen bond

aaRS activity is regulated by various interactions between amino acids, such as hydrogen bonds, hydrophobic interactions, salt bridges, π-stacking, and metal complexes, and mutating amino acids evoke new results (Kaiser et al. 2020). The Mc TyrRS wt, Mc TyrRS mutant 6 (Y33G, D162T), and mutant Mc TyrRS (Y33G, Y112F, and D162T) structure were predicted using homology modeling to elucidate the mutation’s effect (Fig. 3). Using 3D modeling, we confirmed that all mutations in the mutant Mc TyrRS (Y33G, Y112F, and D162T) transpired in the amino acid binding site of the catalytic domain.

## Discussion

*M. jannaschii* mutant tyrosyl tRNA synthetase and mutant tRNA^Tyr^ pair is widely used for genetic code expansion for various ncAA incorporation in *E. coli* (Young et al. 2010). For the attempt to broaden new orthogonal aaRS pool for genetic code expansion, tyrosyl tRNA synthetase of *M. concilii* GP6 was targeted for development of new orthogonal aaRS according to the highest similarity of previously developed mutant Mj TyrRS. As the Mc TyrRS had highest similarity, it was expected to charging *M. jannaschii* mutant tRNA^Tyr^, which was previously developed to be orthogonal and not to be interrupted by endogenous *E. coli* aaRS (Wang and Schultz 2001).

As the rational approach for substrate recognition, specific three residues of Mc TyrRS (Y33, D162, and L116) were substituted to other amino acids. The activity of mutants was confirmed by expressing sfGFP harboring amber codon and observing cells with fluorescence microscope. sfGFP was expressed with the Mc Tyr wt in the presence and absence of AzF. This meant that Mc Tyr wt could recognize tyrosine or both. However, as a result of mutation, Mc TyrRS mutants 4 ~ 6 did not recognize tyrosine as a substrate showing no fluorescence in the absence of AzF. Furthermore, they were not able to recognize AzF showing no or little fluorescence in the presence of AzF. These results indicated that Mc TyrRS mutants 4 ~ 6 could only recognize tRNA, but not tyrosine and AzF. These results implied that the site-specific mutation was not enough, and another mutation was necessary to develop new aaRS mutants.

To promote the aaRS recognize AzF, random mutagenesis and screening were performed based on the Mc TyrRS mutant 6. As a result, mutant Mc TyrRS (Y33G, Y112F, and D162T) was isolated by random mutagenesis. When the AzF was present, mutant Mc TyrRS displayed similar normalized fluorescence intensity to that of Mc TyrRS wt and it was 80% of the sfGFP wt. Also, when the AzF was absent, mutant Mc TyrRS showed similar value of Mc TyrRS mutant 6, which was not capable of AzF recognition. These results indicated that mutant Mc TyrRS specifically recognized AzF. The selectivity was also quantified by calculating the ratio of normalized fluorescence values dividing the value of presence of AzF to its absence. The selectivity of mutant Mc TyrRS was 3.7, whereas that of the mutant Mj TyrRS was 2.8 representing the mutant Mc TyrRS utilizes AzF more efficiently.

Structural prediction of synthetase was performed to figure out the effect of mutations on Mc TyrRS. In the Mc TyrRS wt, substrate tyrosine was recognized through Y33 and D162 interaction (Fig. 3a). However, in the Mc Tyr mutant 6, tyrosine at position 33 and aspartic acid at position 162 were replaced by glycine and threonine, respectively (Fig. 3b). The Y33G and D162T mutations removed two hydrogen bonds from the hydroxyl group of tyrosine, a natural substrate, mutating Mc TyrRS to disfavor tyrosine. The Y33 and D162 mutations in the Mc TyrRS were at the same position as the Y32 and D158 mutations in Mj TyrRS, and this result was consistent with the previous study where the Y32G and D158R mutations in the mutant Mj TyrRS decreased tyrosine recognition activity by removing hydrogen bonds from tyrosine’s hydroxyl group (Turner et al. 2005). Furthermore, the Y33G mutation offered space for AzF’s azide group since tyrosine’s large side chain was removed through substitution, with glycine harboring a minimal side chain. However, the Mc TyrRS mutant 6 could not recognize AzF, so random mutagenesis was conducted to supplement the rational approach. The resulting mutant, the mutant Mc TyrRS, was obtained by further mutation of Y112F from the Mc TyrRS mutant 6 (Fig. 3c). The mutant Mc TyrRS’s AzF recognition state was predicted using a docking model to elucidate the role of the Y112F mutation. In our docking model, the exact role of the Y112F mutation remains unclear since it does not interact directly with AzF. We hypothesize that the removed hydrogen bond, a result of this mutation, may indirectly influence AzF recognition. This change could free up threonine, altering its interaction with AzF enhancing substrate recognition. It could be reasoned that the binding pocket for AzF was prepared in Mc TyrRS mutant 6, but the channel to the pocket was inhibited. The further mutation of Y112F could open the channel to the pocket and confer the amino-acylation of AzF. However, the limitations of computational modeling, such as challenges in structure prediction or accurately dealing with non-canonical functional groups like the azide group present in AzF, necessitate further experimental investigation. Specifically, crystal structure analysis is needed to confirm this hypothesis and provide a detailed understanding of the impact of the Y112F mutation.

In summary, we developed a novel aminoacyl-tRNA synthetase for incorporating AzF into protein. TyrRS derived from *M. concilii* GP6 was modified to disfavor tyrosine through site-directed mutagenesis, favoring AzF after random mutagenesis. The Mc TyrRS mutant library was screened using FACS for high-efficiency screening. As a result, mutant Mc TyrRS was developed, exhibiting AzF selectivity. Comparatively, wild-type Mc TyrRS only charged tyrosine, and the Mc TyrRS mutant 6 weakly charged AzF. Furthermore, the predicted structure of mutant Mc TyrRS was analyzed to verify that the Y33G and D162T mutations halted tyrosine recognition by removing hydrogen bonds and that the Y112F mutation could recognize AzF. This study suggests that developing a new aaRS to incorporate non-canonical amino acids from new organisms is feasible through rational design and random mutations with high efficiency.

## Data Availability

All authors declare that the data supporting the findings of this study are available within the article. The sequencing data of *Methanosaeta concilii* tyrosyl-tRNA synthetase were deposited at NCBI((https://www.ncbi.nlm.nih.gov/) under accession number (PE011898.1).
